# Human Breast Milk miRNA, Maternal Probiotic Supplementation and Atopic Dermatitis in Offspring

**DOI:** 10.1371/journal.pone.0143496

**Published:** 2015-12-14

**Authors:** Melanie Rae Simpson, Gaute Brede, Jostein Johansen, Roar Johnsen, Ola Storrø, Pål Sætrom, Torbjørn Øien

**Affiliations:** 1 Department of Public Health and General Practice, Norwegian University of Science and Technology, Trondheim, Norway; 2 Department of Cancer Research and Molecular Medicine, Norwegian University of Science and Technology, Trondheim, Norway; 3 Department of Computer and Information Science, Norwegian University of Science and Technology, Trondheim, Norway; Emory University School of Medicine, UNITED STATES

## Abstract

**Background:**

Perinatal probiotic ingestion has been shown to prevent atopic dermatitis (AD) in infancy in a number of randomised trials. The Probiotics in the Prevention of Allergy among Children in Trondheim (ProPACT) trial involved a probiotic supplementation regime given solely to mothers in the perinatal period and demonstrated a ~40% relative risk reduction in the cumulative incidence of AD at 2 years of age. However, the mechanisms behind this effect are incompletely understood. Micro-RNAs (miRNA) are abundant in mammalian milk and may influence the developing gastrointestinal and immune systems of newborn infants. The objectives of this study were to describe the miRNA profile of human breast milk, and to investigate breast milk miRNAs as possible mediators of the observed preventative effect of probiotics.

**Methods:**

Small RNA sequencing was conducted on samples collected 3 months postpartum from 54 women participating in the ProPACT trial. Differential expression of miRNA was assessed for the probiotic vs placebo and AD vs non-AD groups. The results were further analysed using functional prediction techniques.

**Results:**

Human breast milk samples contain a relatively stable core group of highly expressed miRNAs, including miR-148a-3p, miR-22-3p, miR-30d-5p, let-7b-5p and miR-200a-3p. Functional analysis of these miRNAs revealed enrichment in a broad range of biological processes and molecular functions. Although several miRNAs were found to be differentially expressed on comparison of the probiotic vs placebo and AD vs non-AD groups, none had an acceptable false discovery rate and their biological significance in the development of AD is not immediately apparent from their predicted functional consequences.

**Conclusion:**

Whilst breast milk miRNAs have the potential to be active in a diverse range of tissues and biological process, individual miRNAs in breast milk 3 months postpartum are unlikely to play a major role in the prevention of atopic dermatitis in infancy by probiotics ingestion in the perinatal period.

**Trial Registration:**

ClinicalTrials.gov NCT00159523

## Introduction

Breast milk is first and foremost a source of nutrition for the newborn infant. However, it is well established that breast milk also provides a direct immune defence against pathogens through immune cells and molecules such as immunoglobulin and lysozymes.[1–3] Additionally, observational studies have demonstrated a negative association between breastfeeding and the development of immune related diseases, including type 1 diabetes mellitus, ulcerous colitis and coeliac disease[4, 5]. This suggests that breastfeeding also has long lasting consequences through its early influence on the developing immune system. Allergy related diseases, such as atopic dermatitis (AD), asthma and allergic rhinoconjunctivitis (ARC), are also considered to be a result of an altered immune system development during infancy, however the association between breastfeeding and these diseases remains controversial.[3] Various components of breast milk have been suggested to contribute to its long term immunological effects, including growth factors, cytokines and more recently microRNAs.

MicroRNAs are a group of short, non-coding, RNA molecules (~22 nucleotides) that regulate gene expression at the post-transcriptional level.[6, 7] Extracellular miRNAs have been identified in several body fluids including serum, breast milk, amniotic fluid and urine.[8] These miRNAs are protected from RNase activity through their association with extracellular vesicles and proteins, such as Argonaute-2 (Ago2)[9], and they have come under particular attention because of their potential role in intercellular communication. Compared to other body fluids, breast milk has a large quantity of total-RNA[8], and a high proportion of miRNAs in breast milk are considered to be “immune-related”.[8, 10–12] This has led to the hypothesis that breast milk miRNAs are one of the mechanisms that breastfeeding affects the early development of an infant’s immune system. In support of the biological plausibility of this hypothesis, *in vitro* studies have demonstrated that breast milk miRNAs are stable under a variety of harsh conditions, including prolonged exposure to acidic solutions simulating the stomach environment[10, 11], and animal studies have suggested that the miRNA expression in milk is correlated with that of serum samples from suckling infants.[13, 14]

The breast milk samples analysed in this study were collected during the Probiotics in the Prevention of Allergy among Children in Trondheim (ProPACT) trial, which demonstrated that perinatal ingestion of probiotics by mothers reduced the cumulative incidence of AD by 40% at 2 years of age.[15] This is a reproducible finding with two meta-analyses demonstrating a beneficial effect of maternal and or infant probiotic supplementation in the primary prevention of AD.[16, 17] These meta-analyses also indicate that postnatal supplementation is necessary to prevent AD, yet prenatal supplementation appears to strengthen the preventative effect. The biological mechanisms which mediate these effects are incompletely understood. In the current study we investigated the possibility that the prevention of AD is partially mediated by alterations in breast milk miRNAs.

The aim of this study was three-fold: first, to determine the miRNA profile of human breast milk samples in the largest collection of human milk samples to date, second, to examine if this profile is influenced by maternal probiotic supplementation and third, to assess if any changes in the miRNA profile are associated with the development of AD in offspring.

## Methods

### Participant recruitment

Breast milk samples were collected from women who participated in the ProPACT trial, a placebo controlled, randomised trial investigating the effect of maternal probiotic supplementation on the development of allergic diseases in early childhood as described elsewhere.[15] Briefly, 415 pregnant women living in Trondheim, Norway, and who intended to breastfeed were randomised to receive equivalently tasting study milk containing probiotic bacteria or sterile cultured milk as a placebo from 36 weeks gestation until 3 months postnatal. The probiotic milk contained 5 x 10^10^ colony-forming units (CFUs) of *Lactobacillus rhamnosus GG* (LGG) and *Bifidobacterium animalis* subsp. *lactis* Bb-12 (Bb-12) and 5 x 10^9^ CFU of *Lactobacillus acidophilus* La-5 (La-5) per day. Questionnaires detailing dietary habits, living conditions and symptoms of allergic disease were completed during pregnancy and 6 weeks, 12 months and 2 years postpartum. AD was diagnosed by a paediatrician at the 2 year clinical examination in accordance with the UK Working Party’s diagnostic criteria for AD.[18]

**Fig 1:**
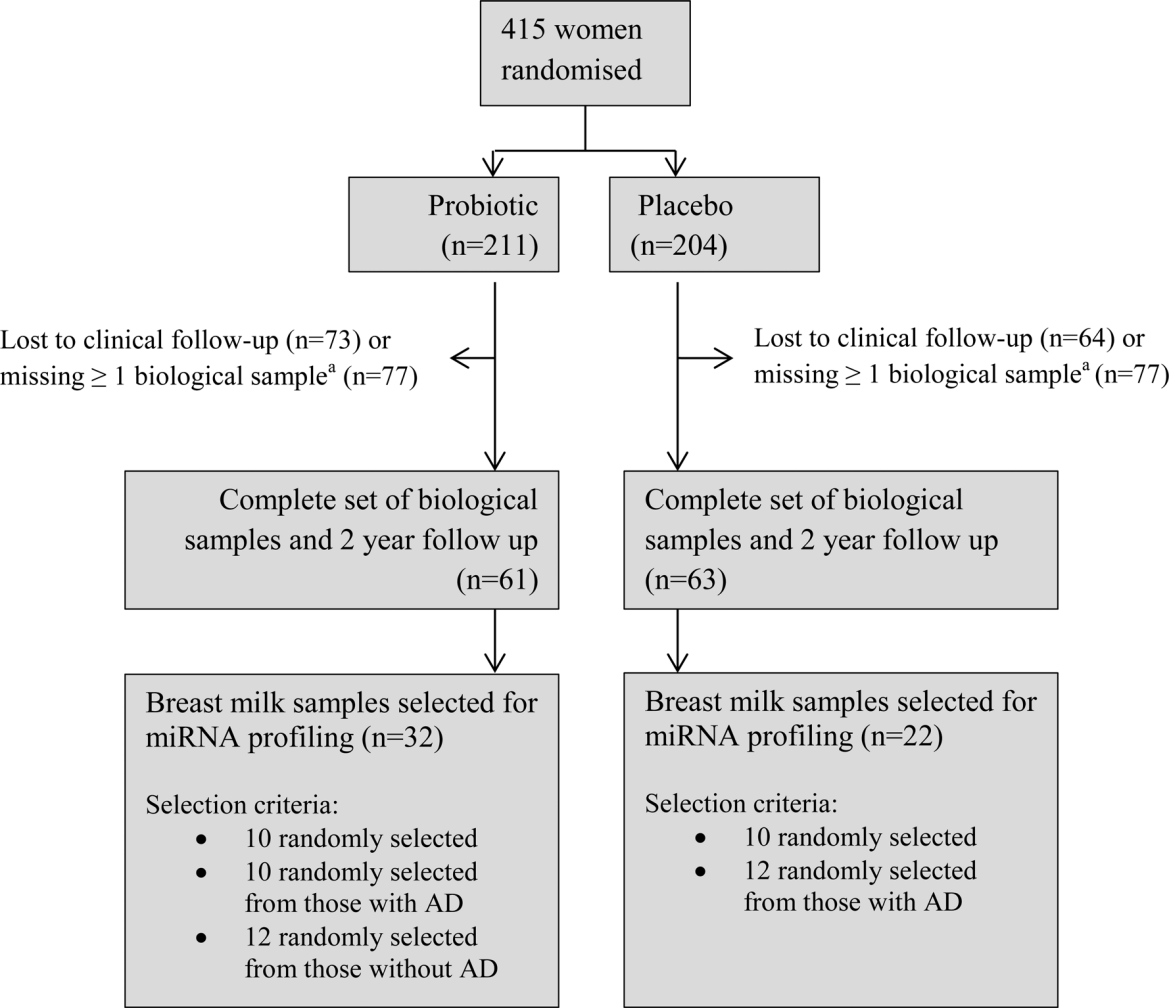
Patient flow and sample selection for ProPACT trial and miRNA sequencing project. ^a^Missing 1 or more of the following biological samples collected at 3 months post-partum: breast milk and stool samples from mother, blood and stool samples from the infant.

To maximise the available information, mother-infant pairs were only eligible to be included in the current study if the child attended the 2 year clinical examination and the following biological samples collected 3 months postpartum were available: breast milk from the mother, a blood sample from the infant and stool samples from both. There were 124 eligible mother-infant pairs, from which 54 breast milk samples were randomly selected according to the criteria in Fig 1.

### Breast milk sample collection

Participants were requested to collect breast milk samples at 10 days and 3 months post-partum. Only the 3 month samples were used in this analysis. Samples were collected into a sterile test container and frozen immediately in the participant’s home freezer until transportation to the laboratory, where they were stored at -80°C until analysis. Length of storage ranged from 7–9 years. The timing of collection with respect to time of day and phase of lactation, ie whether fore- or hind-milk was collected, was not standardised.

### Extracellular vesicle isolation

After thawing on ice, 1.5 mL of whole milk was centrifuged at 2000g for 15 mins at 4°C. The aqueous portion beneath the fat layer was aspirated and centrifuged twice at 16,000g for 40 mins and then 60 mins at 4°C. Subsequently, 500μL of the cell- and debris-free, defatted breast milk obtained after the third centrifugation was mixed with 250μL of ExoQuick Exosome Precipitation Solution™ (System Biosciences, CA, USA) and refrigerated for 12–14 hours at 4°C. The breast milk extracellular vesicles precipitated by this solution were pelleted at 1500g for 30mins at 4°C and resuspended in 100μL of RNAse free water. The breast milk extracellular vesicles solution was used immediately in the RNA isolation process.

### RNA isolation and quantification

The breast milk extracellular vesicles were lysed in 500μl QIAzol solution (Qiagen) for 5 mins at room temperature, and RNA isolation was conducted using the Qaigen miRNeasy kit, as per the manufacturer’s instructions and without the optional Buffer RWT and second Buffer RPE washing steps. To ensure all RNA fragments were collected, the isolated RNA was eluted twice using 50μl RNase-free each time. The concentration of total RNA was measured using a NanoDrop 1000 instrument (Thermo Scientific, Wilmington, USA) and selected samples were analysed with Agilent Bioanalyzer 2100 using the Agilent RNA 6000 Nano Kit for total RNA and Small RNA kit for a focused review of the small RNA.

### Sequencing and bioinformatics pipeline

Small RNA sequencing was conducted by Ocean Ridge Biosciences (ORB, Palm Beach Gardens, FL). Library construction was performed using the ScriptMiner™ Small RNA-Seq Library Preparation Kit (Epicentre Biotechnologies, Madison, WI) on RNA fragments of 11–28 nucleotides (nt) in length from the total RNA from each sample (for more details see S1 Protocol). The samples were sequenced using 50-base pairs (bp) single-end reads on the HiSeq 2000 sequencing system (Illumina Inc, San Diego, CA). Raw reads were processed with cutadapt.[19] Low-quality bases (<20) were removed from reads before adapter removal and the final reads were required to have a length of at least 17 bp. Mapping of reads to the human genome was done with STAR version 2.4.0[20], requiring a perfect match alignment. featureCounts version 1.4.0[21] was then used to count every hit of miRNAs in miRBase version 20, and finally a count matrix was produced by local scripting.

### Statistical analysis and functional predictions

All statistical analyses were conducted in R version 3.03[22]. miRNA read counts were standardised and assessed for differential expression using the voom[23] and limma[24, 25] packages, respectively. Due to the varying proportions of miRNA in each sample, the mature miRNA reads were normalised to the total number of reads matched to mature miRNAs to create a count per million (cpm) value for each miRNA. Within the limma package, a linear model[25] was fitted to the data based on treatment allocation and development of AD. Subsequently, comparisons were made to assess the marginal effect of probiotic treatment and AD development on the expression levels for each miRNA. Comparisons were limited to miRNAs which had an expression level of ≥500 cpm in ≥4 samples (n = 125) in order to increase the likelihood of identifying biologically significant differences. The marginal effects of treatment allocation and AD diagnosis were also assessed in an alternate model which included maternal atopy and the presence of older siblings as covariates. A raw p-value of ≤0.05 was considered of potential interest and false discovery rate (FDR) was controlled for using the Benjamini-Hochberg method[26] with a FDR of ≤0.05 being considered acceptable.

Potential target genes were predicted for the 20 most highly expressed miRNAs and each of the differentially expressed miRNA lists using a locally executed TargetScan version 7.0 algorithm[27–30] with an upper threshold for the context score at -0.2. The lists of unique target genes were subsequently uploaded to the Database for Annotation, Visualization and Integrated Discovery (DAVID) v6.7[31, 32] to gain insight into potential functional consequences of these miRNAs through review of the functional annotation clusters and pathways enrichment using DAVID defined defaults for annotation categories. Additional functional annotation analysis was conducted on the subset of genes targeted by the 20 most highly expressed miRNAs and which were identified as up-regulated in epithelial tissue in the “UP_TISSUE” chart under the tissue expression category in DAVID. When few target genes were identified, they were assessed individually using the DAVID annotation table.

### Trial registration and ethical approval

The clinical trial and this sequencing study were approved by the Regional Committee for Medical Research Ethics for Central Norway (Ref. 097–03) and written consent was obtained from the participating families. The original trial protocol is registered in ClinicalTrials.gov (identifier NCT00159523). The knowledge and technology required for this miRNA analysis was not available when the ProPACT trial commenced and thus is not described in the protocol.

## Results

### Participants

Fifty-four mother-infant pairs were included in this analysis, of which 32 had been randomised to receive probiotic milk, and 22 received placebo milk. The baseline characteristics and the clinical outcomes of the mother-infant pairs are provided in Table 1. There was no substantial difference between the treatment groups with respect to maternal age, gestational age at birth, birth weight, or the presence of older siblings. The probiotic group had a higher proportion of male infants, a difference also observed in the total ProPACT population[15], and a lower proportion of participants with a family or maternal history of atopy, due to the selection criteria (Fig 1).

**Table 1:** Baseline characteristics and clinical outcomes for mother-infant pairs.

	Probiotic group (n = 32)	Placebo group (n = 22)
**Mother-infant pair baseline characteristics:**		
Age, mother (years), mean (SD)	30.51 (4.17)	31.18 (4.44)
Gestational age (days), mean (SD)	283.1 (10.51)	281.2 (14.54)
Birth weight (g), mean (SD)	3581 (402)	3537 (449)
Gender (male), n (%)	18 (56.3)	10 (45.5)
Premature^a^, n (%)	0 (0.0)	2 (9.5)^b^
No siblings, n (%)	17 (53.1)	13 (59.1)
Atopy in family, n (%)	19 (59.4)	16 (72.7)^b^
Maternal atopy, n (%)	11 (34.4)	14 (63.6)
**Post-randomisation characteristics** ^**b**^:		
Breastfeeding		
At least 3 mo., n (%)	31 (96.9)	21 (100)
Duration exclusive, mo., med (range)	4 (0–7)	5 (1–8)
Age of weaning, mo., med (range)	12 (2–24)	11 (4–22)
Sample collection, days (SD)	89.5 (8.1)	93.7 (15.5)
Age of samples, years (SD)	8.4 (0.6)	8.5 (0.6)
**Infant clinical outcomes at 2 years of age:**		
Atopic dermatitis, n (%)	11 (34.4)	18 (81.8)
IgE associated, n (%)	5 (15.6)	3 (13.6)
Non-IgE associated, n (%)	6 (18.8)	14 (63.6)
Sensitisation, n (%)	7 (21.9)	3 (13.6)
Asthma, n (%)	2 (6.3)	2 (9.1)
Allergic rhinitis, n (%)	1 (3.1)	0 (0.0)

^**a**^Defined as birth before 37 weeks gestation

^b^missing information for some individuals

All mothers reported compliance with the research milk consumption from birth until 3 months postpartum and all but two were also compliant prenatally. None of the women included in the current study reported consumption of other probiotic supplements or probiotic enriched products.

### Small RNA profile of human breast milk

**Fig 2:**
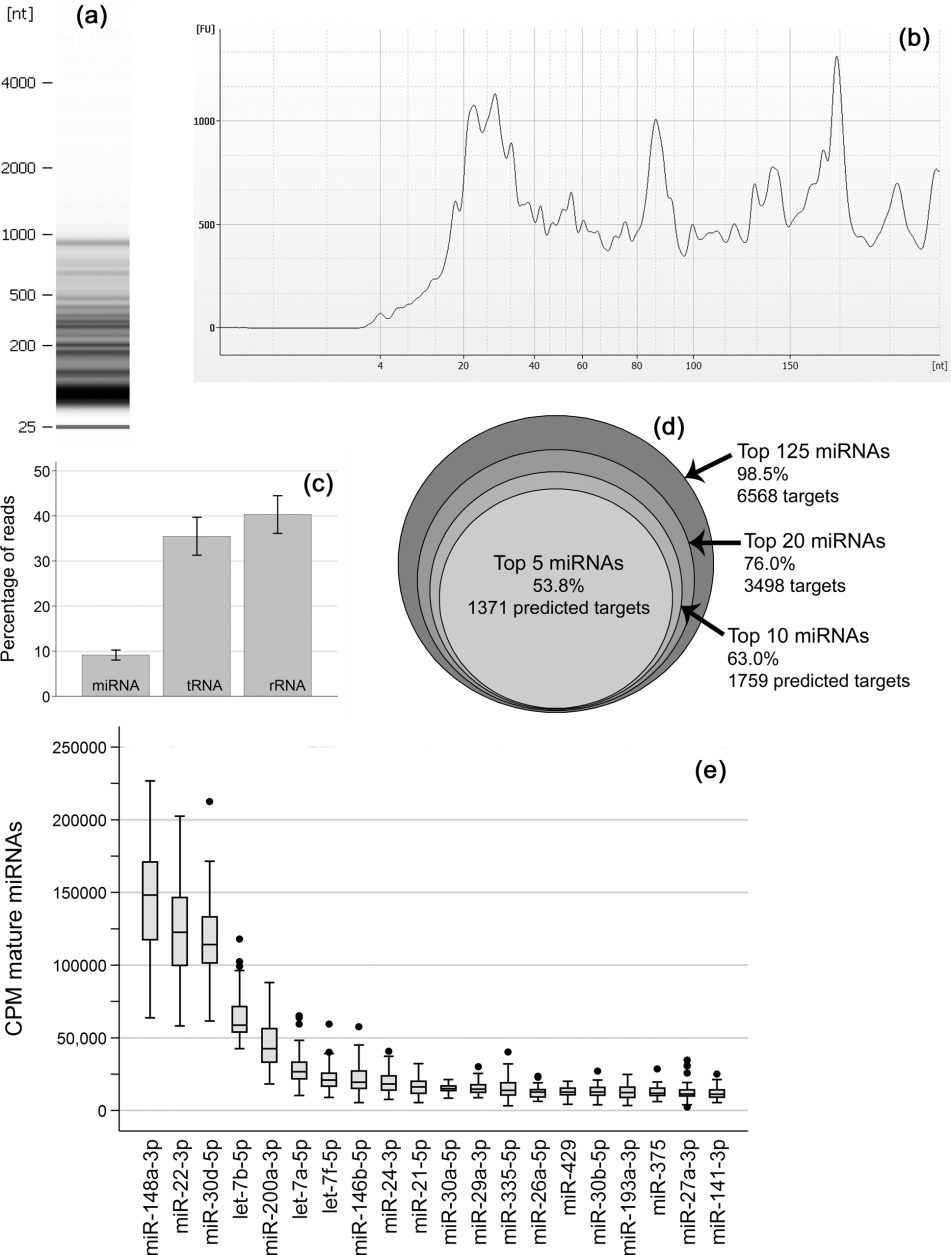
Overview of RNA profile and relative abundance of highly expressed miRNAs from breast milk samples. (A) Bioanalyzer 6000 Nano gel from a representative sample showing abundant short RNAs up to 1000nt; (B) closer review of small RNAs using Agilent’s Small RNA kit demonstrating peaks at 22-23nt, 29-30nt, 33-34nt, 53-54nt, 90nt and approximately 140nt and 170nt; (C) bar graph demonstrating average percentage of small RNA sequences aligned to different RNA species with 95% confidence intervals; (D) proportion of reads accounted for by the top 5, 10, 20 and 125 miRNAs along with the number of predicted target genes (excluding repeated target prediction for alternate transcripts of the same gene); (E) boxplot of counts per million (CPM) mature miRNA of the 20 most abundant miRNAs in the 54 samples.

The RNA isolates contained between 12.4 and 247.5 ng/μL of total RNA. Bioanalyzer analysis indicated that there exists significant amounts of RNA up to 1000 nt in length (Fig 2A) and focused analysis of small RNAs revealed concentration peaks at 22–23, 29–30, 33–34, 53–54, 90 and ~140 and 170 nt (Fig 2B). Small RNA sequencing of the 54 breast milk samples resulted in 1,938,162,564 raw reads with median number 34.7 million (range 17.3 M -163 M).

After trimming of low-sequence bases and adapter removal there were 1.3 billion reads left. Approximately 94.8% (1.25 billion) of these reads were perfectly matched to the human genome (hg 19 version) and used in subsequent analysis. Sequences were aligned to several different RNA species including ribosomal RNA (rRNA), transfer RNA (tRNA), mature miRNA, and other RNAs (Fig 2C). There was no association between the proportion of total reads aligned to mature miRNA sequences and treatment group allocation or AD development in the children (data not shown).

### miRNA profile of human breast milk and functional predictions

The average proportion of reads aligned to mature miRNA species was 9.1% (SD 3.82%, range 2.75–20.16%). The top 5 miRNAs were consistently highly expressed and included miR-148a-3p, miR-22-3p, miR-30d-5p, let-7b-5p and miR-200a-3p. These miRNAs accounted for 54% of all mature miRNAs (Fig 2D and 2E and S1 File). The miRNAs ranked 6^th^ to 20^th^ by overall mean expression showed a greater variability in individual ranking. Using these 20 most abundant miRNAs, 3498 unique potential gene targets were identified with the TargetScan algorithm. Functional annotation clusters of these predicted targets indicate that the breast milk miRNAs may have the greatest effect on a) the positive and negative regulation of metabolic processes involving nitrogen compounds, RNA, DNA and macromolecules and the positive regulation of transcription and gene expression, b) embryonic development, c) angiogenesis, d) catabolic processes and e) cell migration and localisation. The full results of the functional annotation clustering analysis, gene ontology and pathway analysis conducted in DAVID is provided in S2 File.

To date, it is unknown if, and in which tissues, breast milk miRNAs are active after ingestion. Based on tissues identified by DAVID to have upregulated expression of the predicted target genes, these miRNAs as a group have the potential to be most biologically influential in the brain (FDR = 3.32 x 10^−31^) and epithelium (FDR = 5.01 x 10^−12^). Other tissues where this gene set is highly expressed include female reproductive tissues, such as placenta, uterus and endometrium, foetal tissues, such as the foetal brain, kidney and lung, and haemopoetically involved tissues, such as platelets, T-cells, bone marrow, fibroblasts, and the thalamus (UP_TISSUE sheet in S2 File).

Functional clustering analysis conducted on the subset of predicated target genes identified as highly expressed in epithelial cells revealed that genes with activities located in the non-membrane-bounded organelles, cytoskeleton and nucleus were significantly enriched. The most highly enriched biological processes and molecular functions included chromatin and chromosome organisation, transcription and negative regulation of gene expression and biosynthetic processes (S3 File). The breast milk miRNA profile may also represent a beneficial collection of miRNAs for mammary gland development and or maintenance during lactation and in this context the mammary gland epithelium may also be a site of action. Healthy mammary tissues were otherwise not identified as being highly enriched with the potential target genes (UP_TISSUE sheet in S2 File).

### miRNA profiles associated with maternal probiotic ingestion

Maternal probiotic supplementation was associated with upregulation of let-7d-3p and downregulation of miR-574-3p, miR-340-5p and miR-218-5p, although the estimated FDR for each is 0.818 (Table 2). Furthermore, these miRNAs showed relatively low levels of expression constituting 0.03 to 0.2% of mature miRNAs on average. Together they had 495 predicted target genes. Functional annotation clustering revealed no cluster directly involved with atopic dermatitis, asthma or allergic rhinoconjunctivitis (S4 File). On review of individual targets, the suppressor of cytokine signalling 3 (*SOCS3*) gene was found to be associated with atopic dermatitis in the Online Mendelian Inheritance in Man (OMIM) database. Other identified targets which may be related to the development of allergy related disease through their association with T cell differentiation and activation or transforming growth factor β (TGF-β) are listed in S4 File. These results were unaltered when maternal atopy and presence of siblings were included as covariates (data not shown).

**Table 2:** Differentially expressed miRNAs.

miRNA	Fold change	p-value	FDR
**Probiotic vs Placebo**			
miR-574-3p	0.640	0.016	0.818
miR-340-5p	0.697	0.040	0.818
let-7d-3p	1.401	0.044	0.818
miR-218-5p	0.690	0.050	0.818
**Atopic dermatitis vs no-atopic dermatitis**			
miR-452-5p	0.660	0.001	0.107
let-7d-3p	1.615	0.005	0.308
miR-146b-5p	0.674	0.011	0.433
miR-21-5p	0.752	0.016	0.433
miR-22-3p	1.258	0.019	0.433
miR-375	1.247	0.023	0.433
miR-16-5p	0.686	0.026	0.433
miR-511-5p	1.323	0.028	0.433
miR-26b-5p	0.808	0.041	0.461
let-7f-5p	0.802	0.041	0.461
miR-30e-5p	0.844	0.042	0.461
miR-374a-5p	0.797	0.044	0.461
miR-335-5p	1.343	0.049	0.468

### miRNA profiles associated with infant atopic dermatitis

The development of AD by 2 years of age was associated with differential expression of several miRNAs (Table 2). None of these miRNAs had an acceptable FDR and were unaffected by the inclusion of additional covariates. However, a number of them are relatively highly expressed including miR-146b-5p, miR-21-5p, miR-22-3p, miR-375 and let-7f-5p. Functional analysis of the 2269 predicted target genes indicated that these genes were enriched in a diverse range of functional clusters from embryonic development to positive and negative regulation of metabolic processes involving RNA, macromolecules and of transcription and gene expression (S5 File). These functional clusters were particularly related to predicted targets of down-regulated breast milk miRNAs.

## Discussion

In this study we present the miRNA sequencing results from breast milk samples collected 3 months postpartum from 32 women who received probiotic milk and 22 who received placebo milk during the ProPACT trial.[15] Considering first the general miRNA profile of these samples, we found a relatively stable core group of highly expressed miRNAs which are predicted to have a wide range of potential biological effects. We also investigated the effect of maternal probiotic ingestion on the relative abundance of breast milk miRNAs and the association of individual miRNAs with the development of AD. On both of these accounts we observed no conclusive evidence of differentially expressed miRNAs. As such, individual breast milk miRNAs at 3 months postpartum are unlikely to play a major role in the mechanism behind the observed preventative effect of perinatal probiotics on AD.[15] Before further discussing the main findings, we first highlight the strengths and limitations of this study.

The major strength of this study is that it is the largest sequencing analysis of human milk small RNAs to date, allowing characterisation of the general profile of breast milk miRNAs at 3 months postpartum. In using a single collection time point we did not need to consider the temporal variation in breast milk miRNA[10, 13, 33, 34]. We were also able to investigate the relationship between miRNA expression levels and maternal probiotic ingestion and the development of AD in offspring because of the underlying RCT design. For this purpose, we acknowledge that one of the weaknesses of this study is that the sample size only allows us to detect large differences in expression levels. Furthermore, the sample selection criteria favoured the inclusion of mother-infant pairs where the infant had developed AD and there was a maternal or familial history of allergy related disease, particularly in the placebo group. We fitted an alternate model, which included maternal atopy and siblings as covariates, to investigate if the selection procedure had affected the results. The miRNAs identified as differentially expressed in association with treatment allocation and AD development were unchanged in this alternate model. Information regarding maternal antibiotic use was not recorded, however we do not believe that the lack of this information has substantially affected our conclusions.

Other weaknesses and methodological limitations of this study include the age of the samples, extracellular vesicle isolation, and functional analysis methods. The breast milk samples had been stored for between 7 and 9 years prior to analysis and, although breast milk miRNAs are reported to be stable, it is unclear whether prolonged storage alters the general miRNA profile of human milk. Reassuringly, we found no relationship between the amount of RNA isolated and the age of the samples (data not shown). Recent methodological papers suggest that our extracellular vesicle isolation method would have maximised the quantity of RNA isolated[35], but in doing so has captured protein-miRNA complexes which are both extracellular and extravesicular[35], as well as extracellular vesicles released by lysed maternal cells upon freezing.[36] The current article is primarily interested in the biologically available miRNAs in human breast milk and we consider our isolation procedure to be a reasonable approach given infants ingest breast milk in its entirety, extracellular vesicles, protein-miRNA complexes and maternal cells included. In support of this thinking, Gu et al[13] demonstrated relationships between several porcine milk miRNAs and their serum levels in suckling piglets using near identical preparation methods on previously frozen samples. Whilst Gu et al’s findings imply that milk miRNAs are biologically available after ingestion, the investigation of the functional consequences of milk miRNAs is complicated and the available functional prediction tools have certain limitations. Specifically, gene targets are predicted rather than validated and no available tools simultaneously account for the relative abundance of the miRNAs, the number of miRNA-target interactions for any given miRNA or target, and the downstream effects of these interactions. We consider our functional annotation results to be speculative. All the same, evidence from experimental and animal studies suggests that breast milk miRNAs are stable[10, 11] and biologically active after ingestion[13, 14], which opens up the possibility that they may be involved in maternal guidance of the developing immune system and gastrointestinal tract.

The five most abundant miRNAs identified in the breast milk samples were miR-148a-3p, miR-22-3p, miR-30d-5p, let-7b-5p and miR-200a-3p. These 5 miRNAs were consistently highly expressed in all samples whilst other miRNAs showed greater variability in their relative ranking and are perhaps more influenced by individual characteristics, such as genetics, age, parity, diet or other environmental factors. Previous studies of human milk miRNA have also observed that a few highly expressed miRNAs are responsible for the majority of miRNA counts[11, 12], yet the list over the top 10 or 20 miRNAs varies between studies (Table 3).[8, 10–12] It would appear that the miRNA profile is particularly influenced by the miRNA quantification method, and may also depend on the milk fraction used for RNA isolation, time postpartum, ethnicity of the women and the dietary or environmental exposures of their cultures, and the bioinformatics pipeline used for filtering and alignment. Notably, the two sequencing analyses by Zhou et al[11] and Munch et al[12] showed the greatest similarity to the profile we observed despite the varying ethnic backgrounds. This would suggest that our results are generalisable beyond the predominantly Caucasian population included in the ProPACT study. Interestingly, the study by Munch et al[12] isolated miRNA from the lipid fraction of breast milk, yet observed a very similar miRNA profile to the extracellular vesicle enriched milk samples of the current study and Zhou et al[11]. The biological significance of this observation is uncertain, however it may suggest that even if miRNAs are preferentially selected into breast milk, their inclusion into extracellular vesicles or association with the lipid fraction may be less important for their biological actions.

**Table 3:** Comparison of timing, methods and results of the current study and previous studies of human breast milk miRNA.

Author (year)	n	Time postpartum	Method miRNA quantification	Breast milk portion	Comments and most highly expressed miRNAs^a^
Current study	54	3 mths	Illumina RNA seq, 50bp single-end reads	EV enrichment using Exoquick	Norwegian women participating in RCT investigating probiotics in prevention of allergy related diseases. Top 10 miRNAs: **miR-148a-3p, miR-22-3p, miR-30d-5p, let-7b-5p, miR-200a-3p, let-7a-5p, let-7f-5p, miR-146b-5p, miR-24-3p, miR-21-5p**
Zhou (2011)	4	60 days	Illumina RNA seq, 36bp single-end reads	EV enrichment using Exoquick	Chinese women. Top 10 miRNAs: **miR-148a-3p, miR-30b-5p, let-7f-5p, miR-146b-5p, miR-29a-3p, let-7a-5p, miR-141-3p**, miR-182-5p, **miR-200a-3p**, miR-378-3p.
Munch (2013)	3	6–12 weeks	Illumina RNA seq, 36bp single-end reads	Lipid fraction	Two (2) Breast milk samples were sequenced from 3 American of varying ethnic backgrounds. Study participants underwent pharmacological stimulation of breast milk production and submitted multiple samples at 3 hourly intervals. Top 10 miRNAs^b^: **mir-148a-3p, let-7a-5p,** mir-200c-3p, **mir-146b-5p**, **let-7f-5p**, **mir-30d-5p**, mir-103a-3p, let-7b-5p, let-7g-5p, **mir-21-5p**.
Kosaka (2010)	8	2–11 mths	MicroRNA microarray (Agilent)	Defatted, cell and debris free milk	Eight (8) Japanese women submitted up to 4 samples at varying time points. No quantitative results published beyond those for miRNA considered to be “immune related”. Reported immune related miRNA with high expression^c^: miR-92a-3p, miR-155-5p, miR-181a-5p, miR-181b-5p, let-7i-5p, **miR-146b-5p**, miR-223-3p, miR-17-5p
Weber (2010)	5	Not reported	miScript Assay (incl. 714 miRNA produced by Qaigen)	Defatted, cell and debris free milk	Commercially available samples from 5 “healthy” women. Ethnicity and timing of sample collection is unspecified. Top 10 miRNAs^d^: miR-335-3p, miR-26a-2-3p, miR-181d-5p, miR-509-5p, miR-524-5p, miR-137, miR-26a-1-3p, miR-595, miR-580-3p, miR-130a-3p

EV: extracellular vesicles

^a^miRNAs in bold type were also found within the top 20 miRNAs of the current experiment

^b^miRNA names are converted to miRBase version 21.0 annotation version 16.0

^c^miRNA names converted from unspecified earlier version

^d^miRNA names converted from miRBase version 13.0. The miRNA names supplied in the study by Zhou et al (2011) required no conversion.

The functional analysis of the breast milk miRNA profile revealed that the highly expressed miRNAs are potentially involved in the regulation of genes in many transcriptional, metabolic and biosynthetic processes. The ultimate effect of the cooperative regulation by breast milk miRNAs is difficult to decipher from this analysis. We have considered the intestinal epithelium to be the most likely first site of action in the infant and in support of this theory epithelial tissues were identified as having a gene enrichment profile that significantly overlaps with the predicted gene targets for the top 20 breast milk miRNAs. Following the birth of a healthy term infant, the intestinal tract matures resulting in a less permeable epithelium.[37] The early establishment of a well-functioning intestinal barrier is thought to be integral in the normal development of the immune system and a defective intestinal barrier in early infancy has been linked to several infant gastrointestinal diseases and a predisposition to autoimmune and inflammatory diseases later in life, including atopic dermatitis.[37] Breast milk appears to assist this maturation process through growth factors, hormones and cytokines, and studies have shown breastfeeding to be associated with reduced intestinal permeability[37, 38], morphological maturation[39, 40] and altered intestinal gene expression[41] in the early neonatal period. Additionally, the intestinal microbiota composition and diversity is also considered to be involved in promoting intestinal maturity and breast milk is thought to promote a “healthy” microbiota by transferring microbes, prebiotic milk oligosaccharides and by encouraging an anti-inflammatory, environment allowing microbial tolerance.[38, 42] We propose that breast milk miRNAs may be another factor contributing to intestinal maturation and microbiome establishment. Experimental evidence in support of this theory comes from mouse studies which have demonstrated improved epithelial barrier function associated with miR-146b[43], involvement of miR-375 in epithelium-immune system crosstalk[44] and promotion of innate immune tolerance in the neonatal period by miR-146a[45]. Although miR-146a was not highly expressed in breast milk samples, the more highly expressed miR-146b also targets interleukin 1 receptor associated kinase 1 (*IRAK1*), a Toll-like receptor (TLR) signalling molecule thought to be involved in the miR-146a mediated promotion of immune tolerance. Unfortunately, it is difficult to isolate the effects of breast milk miRNAs from other biologically active components of breast milk. In a pig experiment, Hu et al[40] found that weaning was associated with increased permeability and activation of mitogen-activated protein kinases (MAPK) in piglets, altered morphology and increased permeability. Interestingly, the analysis of gene targets of highly expressed breast milk miRNAs revealed enrichment of the MAPK signalling pathway. The results of Hu et al[40] would be consistent with a loss of a breast milk miRNA mediated down regulation of these pathways after weaning. On the other hand, gene expression analysis of intestinal epithelial cells from breastfed and formula fed human infants reveals that target genes of the top 20 miRNA are both up and down regulated in the breast fed infants[41], indicating that the biological consequences of breast milk miRNAs are either minimal or substantially more complex.

Looking to other mammalian species, there are similarities in the milk miRNA profile of pigs[13, 46], cows[47], rats[48] and tammar wallabies[33]. In particular, miR-148a, miR-30a, let-7a, let-7b and let-7f are reported to be expressed in moderate to high quantities across these species. The conservation of these miRNA, not only in their structural nature, but in their inclusion in breast milk over several mammalian species, implies that they are evolutionarily selected and have beneficial roles for the mother and or her offspring. With this in mind, it is perhaps not surprising that a four month dietary intervention did not convincingly affect the breast milk miRNA profile.

A number of differentially expressed miRNAs were identified on comparison between the probiotic and placebo group and between samples from mothers whose children did and did not develop AD. None of these miRNAs had an acceptable FDR after controlling for multiple comparisons. Maternal probiotic ingestion was associated with four differentially expressed miRNAs with low abundance (miR-574-3p, let-7d-3p, miR-340-5p and miR-218-5p). As such, we found no conclusive evidence to suggest that maternal probiotic ingestion significantly alters the relative abundance of individual miRNAs and is therefore not a major mechanism by which the protective effect of probiotics is conveyed to the newborn infant. On the other hand, a number of breast milk miRNAs found to be associated with the development of atopic dermatitis were the relatively highly expressed miRNAs: miR-22-3p, miR-146b-5p, miR-21-5p, miR-375 and let-7f-5p. Curiously, the upregulation of let-7d-3p and miR-375 and downregulation of miR-21-3p and miR-146b-5p are opposite to what one would have expected based on a previously published review of miRNAs in allergic diseases[49]. It is unclear what the biological significance this observation has in terms of the impact of breast milk miRNAs and the development of allergic disease.

The functional annotation results are speculative and further research will need to investigate the biological availability and function of these miRNAs. It will be particularly useful to establish models to assess the cooperative gene regulation of breast milk miRNAs, and other non-coding RNAs, as a group. Such experiments and bioinformatics models may shed light on the biologically plausibility of a relationship between AD and the miRNAs differentially expressed in association with probiotic ingestion, something which is doubtful from this analysis. This study sequenced samples taken 3 months postpartum, at the end of the intervention period, however colostrum samples taken in the first 10–14 days postpartum may reveal a different profile with greater influence on the development of AD. Another line of future enquiry is to characterise and investigate the function of other short non-coding RNAs, such as fragmented tRNAs, which have also been reported in other extracellular vesicles isolated from other body fluids.[50]

In conclusion, there appears to be a stable group of core breast milk miRNAs, which are at least partially conserved across a number of mammalian species. The biological functions of this, presumably evolutionarily-driven, collection of miRNAs is uncertain. Functional analysis of the potential target genes of highly expressed breast milk miRNAs revealed enrichment in a broad range of biological processes and molecular functions. Although several miRNAs were found to be differentially expressed on comparison of the probiotic and placebo groups and AD vs non-AD, none had an acceptable FDR and their biological significance on the development of AD is not immediately apparent from functional analysis. Future experimental and bioinformatics techniques should investigate the biological consequences of highly and differentially expressed miRNAs as a group.

## Supporting Information

S1 Checklist CONSORT 2010 Checklist. Consolidated Stardards of Reporting Trials checklist. (DOC)

S1 File Expressed miRNA. Read processing and miRNA expression information (XLSX)

S2 File Top20 DAVID results. Highly Expressed breast milk miRNAs: Functional analysis for predicted targets of top 20 miRNA (XLSX)

S3 File Epithelium DAVID results. Functional analysis results of epithelial related genes targeted by highly expressed miRNAs.
(XLSX)

S4 File Probiotic vs placebo DAVID results. Probiotic vs placebo: Functional analysis for differentially expressed breast milk miRNAs
(XLSX)

S5 File AD vs nAD DAVID results. Atopic dermatitis versus no atopic dermatitis: Functional analysis for differentially expressed breast milk miRNAs.
(XLSX)

S1 Protocol Supplementary methods. Additional details for methods.
(DOC)
