# Supplementary material for: Human Breast Milk miRNA, Maternal Probiotic Supplementation and Atopic Dermatitis in Offspring
Source: PLoS One. 2015 Dec 14;10(12):e0143496. doi: 10.1371/journal.pone.0143496 (PMC4682386; doi:10.1371/journal.pone.0143496)
Supplement: S1 Checklist — (DOC) [file pone.0143496.s001.doc]

Comment regarding CONSORT 2010 checklist

Please find attached the CONSORT 2010 checklist for the research article entitled: “Human breast milk miRNA, maternal probiotic supplementation and atopic dermatitis in offspring”. The breast milk samples analysed in this study were collected during a randomised controlled trial (RCT) of maternal probiotic supplementation, the Probiotics in the Prevention of Allergy among Children in Trondheim (ProPACT) trial. The design, implementation and the primary and secondary clinical outcomes from the 2 year following-up of this trial have been previously published (Dotterud et al 2010), and the publication of the 6 year follow-up appeared in *BMC Dermatology* (Simpson et al 2015). All of the items in the CONSORT 2010 checklist are covered by these publications.

Whilst the samples were collected during an RCT, the current manuscript submitted to *PLOS ONE* represents a post-hoc exploratory analysis on a semi-randomised selection of the original participants. As such, we feel that many of the items in the CONSORT 2010 checklist are not as appropriate in the context of the submitted manuscript, and their inclusion would detract from the objectives of the current study. We were unsure if the editorial staff at *PLOS ONE,* and potential reviewers, would agree with this assessment and include the CONSORT 2010 checklist for the sake of completeness. We have the following comments regarding specific CONSORT item numbers:

| Item | Comment |
| --- | --- |
| 1a & 1b | Given this study is based on a semi-randomised selection, we feel it would be misleading to identify the article as a “randomised controlled trial” in the title, and the inclusion of the original trial design in the abstract would detract from the objectives and description of the current study. |
| 6a, 19 | The pre-specified primary and secondary outcomes are published elsewhere. The primary clinical outcome of interest to the current study (atopic dermatitis) and the method of diagnosis is described in the methods section (pg. 6). We feel it is unnecessary to include the other primary (asthma, allergic rhinoconjunctivitis) and secondary (sensitisation) clinical outcomes as they are not relevant to the current study. We also identify the current study as being a post-hoc (pg 10). |
| 7a, 8a, 8b, 9, and 10 | Similar to items 1a and 1b, we feel that the details of how the sample size was calculated and how the original randomisation occurred are less relevant to the current analysis which is a semi-randomised selection. The selection of participants for the current study is clearly outlined in Figure 1. Both the sample size and randomisation details have been previously published. |
| 14a | We feel it is more appropriate to clearly state the age of the samples at the time of analysis, rather than the dates of recruitment. |

References

Dotterud CK et al. Probiotics in pregnant women to prevent allergic disease: a randomised, double-blind trial. *British Journal of Dermatology*. May 2010; 163: p616-623.

Simpson MR et al. Perinatal probiotic supplementation in the prevention of allergy related disease: 6 year follow up of a randomised controlled trial. *BMC Dermatology*. 15:13 doi: 10.1186/s12895-015-0030-1


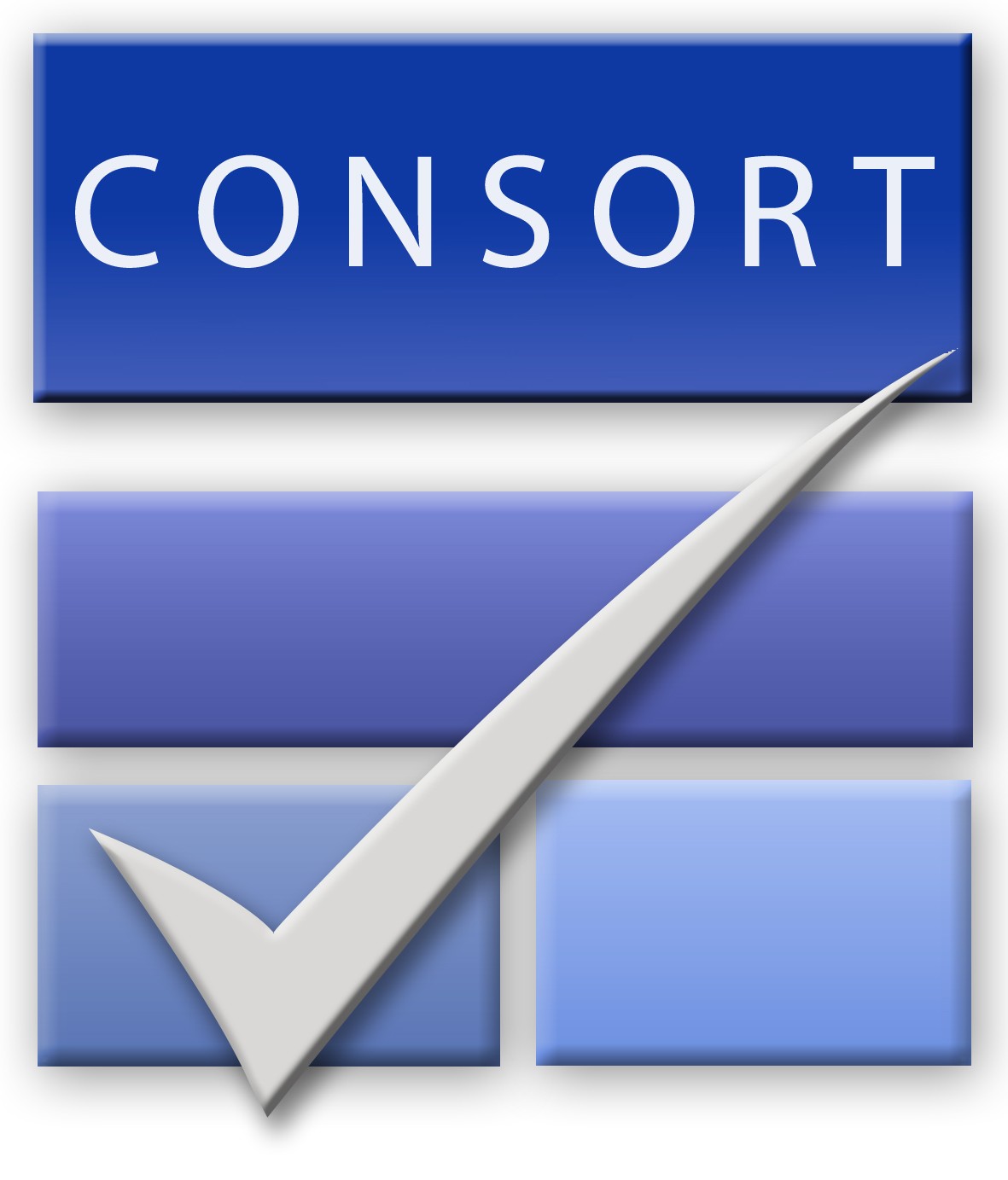
CONSORT 2010 checklist of information to include when reporting a randomised trial*

| Section/Topic | Item No | Checklist item | Reported on page No |
| --- | --- | --- | --- |
| Title and abstract | | | |
|  | 1a | Identification as a randomised trial in the title | See comment above |
|  | 1b | Structured summary of trial design, methods, results, and conclusions (for specific guidance see CONSORT for abstracts) | See comment above |
| Introduction | | | |
| Background and objectives | 2a | Scientific background and explanation of rationale | 4-5 |
|  | 2b | Specific objectives or hypotheses | 4-5 |
| Methods | | | |
| Trial design | 3a | Description of trial design (such as parallel, factorial) including allocation ratio | 6 |
|  | 3b | Important changes to methods after trial commencement (such as eligibility criteria), with reasons | 6 |
| Participants | 4a | Eligibility criteria for participants | 6 |
|  | 4b | Settings and locations where the data were collected | 6-7 |
| Interventions | 5 | The interventions for each group with sufficient details to allow replication, including how and when they were actually administered | 6 |
| Outcomes | 6a | Completely defined pre-specified primary and secondary outcome measures, including how and when they were assessed | See comment |
|  | 6b | Any changes to trial outcomes after the trial commenced, with reasons | 9-10 |
| Sample size | 7a | How sample size was determined | See comment |
|  | 7b | When applicable, explanation of any interim analyses and stopping guidelines | N/A |
| Randomisation: |  |  |  |
| Sequence generation | 8a | Method used to generate the random allocation sequence | See comment |
|  | 8b | Type of randomisation; details of any restriction (such as blocking and block size) | See comment |
| Allocation concealment mechanism | 9 | Mechanism used to implement the random allocation sequence (such as sequentially numbered containers), describing any steps taken to conceal the sequence until interventions were assigned | See comment |
| Implementation | 10 | Who generated the random allocation sequence, who enrolled participants, and who assigned participants to interventions | See comment |
| Blinding | 11a | If done, who was blinded after assignment to interventions (for example, participants, care providers, those assessing outcomes) and how | See comment |
|  | 11b | If relevant, description of the similarity of interventions | 6 |
| Statistical methods | 12a | Statistical methods used to compare groups for primary and secondary outcomes | 8-9 |
|  | 12b | Methods for additional analyses, such as subgroup analyses and adjusted analyses | 8-9 |
| Results | | | |
| Participant flow (a diagram is strongly recommended) | 13a | For each group, the numbers of participants who were randomly assigned, received intended treatment, and were analysed for the primary outcome | Figure 1 |
|  | 13b | For each group, losses and exclusions after randomisation, together with reasons | Figure 1 |
| Recruitment | 14a | Dates defining the periods of recruitment and follow-up | See comment |
|  | 14b | Why the trial ended or was stopped | N/A |
| Baseline data | 15 | A table showing baseline demographic and clinical characteristics for each group | Table 1, p.10 |
| Numbers analysed | 16 | For each group, number of participants (denominator) included in each analysis and whether the analysis was by original assigned groups | Figure 1 |
| Outcomes and estimation | 17a | For each primary and secondary outcome, results for each group, and the estimated effect size and its precision (such as 95% confidence interval) | N/A |
|  | 17b | For binary outcomes, presentation of both absolute and relative effect sizes is recommended | N/A |
| Ancillary analyses | 18 | Results of any other analyses performed, including subgroup analyses and adjusted analyses, distinguishing pre-specified from exploratory | 10-15 |
| Harms | 19 | All important harms or unintended effects in each group (for specific guidance see CONSORT for harms) | See comment |
| Discussion | | | |
| Limitations | 20 | Trial limitations, addressing sources of potential bias, imprecision, and, if relevant, multiplicity of analyses | 17-18 |
| Generalisability | 21 | Generalisability (external validity, applicability) of the trial findings | 18 |
| Interpretation | 22 | Interpretation consistent with results, balancing benefits and harms, and considering other relevant evidence | 15-23 |
| Other information | | |  |
| Registration | 23 | Registration number and name of trial registry | 9 |
| Protocol | 24 | Where the full trial protocol can be accessed, if available | 9 |
| Funding | 25 | Sources of funding and other support (such as supply of drugs), role of funders | Online submission |

*We strongly recommend reading this statement in conjunction with the CONSORT 2010 Explanation and Elaboration for important clarifications on all the items. If relevant, we also recommend reading CONSORT extensions for cluster randomised trials, non-inferiority and equivalence trials, non-pharmacological treatments, herbal interventions, and pragmatic trials. Additional extensions are forthcoming: for those and for up to date references relevant to this checklist, see [www.consort-statement.org](http://www.consort-statement.org/).
