# Supplementary material for: Human Breast Milk miRNA, Maternal Probiotic Supplementation and Atopic Dermatitis in Offspring
Source: PLoS One. 2015 Dec 14;10(12):e0143496. doi: 10.1371/journal.pone.0143496 (PMC4682386; doi:10.1371/journal.pone.0143496)
Supplement: S1 Protocol — Additional details for methods. (DOC) [file pone.0143496.s007.doc]

Supporting Information S1

Additional detail for methods

**Sequencing**

Small RNA sequencing was conducted by Ocean Ridge Biosciences (ORB, Palm Beach Gardens, FL). Samples were treated with RNase-free DNase I and repurified on RNeasy MinElute columns (Qiagen) prior to subsequent sequencing. Sequencing libraries were constructed from the re-purified RNA samples using ScriptMiner™ Small RNA-Seq Library Preparation Kit (Epicentre Biotechnologies, Madison, WI) according to the manufacturer’s instructions with minor modifications. Between RNA linker ligation and cDNA synthesis, RNA was fractionated using 10% acrylamide-urea Gel and RNA fragments of 65-82 nt were excised. This corresponded to reads of 11-28 nt in length and ensured that reads of 17-23 nt were recovered at a minimum and was designed to enrich miRNA reads. Purified libraries were electrophoresed using 8% native polyacrylamide gel and fragments of approximately 122-144 nt were recovered, purified, quality controlled, quantified and pooled at equimolar concentrations.

The samples were sequenced using 50-bp single-end reads using the TruSeq SBS Kit version 3 – HS (Illumina Inc., San Diego, CA) on the HiSeq 2000 sequencing system (Illumina Inc, San Diego, CA). Demultiplexing and production of FASTQ files were conducted using CASAVA software version 1.8. Sequencing was completed within 3.5 months from breast milk extracellular vesicle isolation and RNA isolation.

An initial pilot sequencing project using a wider excision from the 10% acrylamide-urea Gel fractionation step excised fragments of 65-95 nt in length, corresponding to a minimum recovery of reads of 17-30nt, and recovery of library fragments with the approximate size of 122-155 nt after the ScriptMiner protocol. This protocol resulted in a lower proportion of miRNA reads due to abundant tRNA fragments of approximately 30nt (data not shown), and the narrower excision protocol was adopted for the characterisation of miRNA in breast milk.
